# Incidence, healthcare and out-of-pocket costs, and mortality of *Clostridioides difficile* infection among US adults aged 18 to 64 years

**DOI:** 10.1017/ash.2024.400

**Published:** 2024-12-11

**Authors:** Holly Yu, Tamuno Alfred, Jingying Zhou, Jennifer Judy, Margaret A. Olsen

**Affiliations:** 1 Health Economics and Outcomes Research, Pfizer Inc, Collegeville, PA, USA; 2 Statistical Research and Data Science Center, Pfizer Inc, New York, NY, USA; 3 Statistical Data Sciences & Analytics, Pfizer Inc, Peapack, NJ, USA; 4 Evidence Generation Platform, Real World Evidence, Pfizer Inc, New York, NY, USA; 5 Departments of Medicine and Surgery, Washington University School of Medicine, St. Louis, MO, USA

## Abstract

**Objective:**

To estimate incidence and healthcare costs and mortality associated with *Clostridioides difficile* infection (CDI) among adults <65 years old.

**Design:**

Retrospective cohort study.

**Patients:**

First CDI episodes among commercially insured US patients 18–64 years old were identified from a large claims database. CDI+ patients were propensity score−matched (PSM) 1:1 with CDI− controls using clinically relevant variables including comorbidities.

**Methods:**

Annual CDI incidence was calculated by age group and year (2015−2019). Healthcare utilization, costs, and mortality were analyzed by age group, acquisition (healthcare and community), and hospitalization status by calculating CDI-excess costs and mortality as the difference between PSM CDI+ and CDI− individuals.

**Results:**

In 50–64- and 18–49-year-olds, respective CDI incidence per 100,000 person-years decreased from 217 and 113 cases in 2015 to 167 and 87 cases in 2019. Most cases (76.5%–86.9%) were community-associated. The costs and mortality analyses included 6,332 matched CDI+/− 50–64-year-olds and 6,667 CDI+/− 18–49-year-olds. Among 50–64-year-olds, mean 2-month healthcare and patients’ out-of-pocket costs were $11,634 and $573 higher, respectively, in the CDI+ versus CDI− group. Among 18–49-year-olds, 2-month costs were $7,826 and $642 higher. Healthcare costs were higher for healthcare- versus community-associated CDI. At the 12-month follow-up, mortality was significantly higher in the CDI+ versus CDI− groups for both 50–64-year-olds (4.2% vs 2.0%; *P* < .001) and 18–49-year-olds (1.2% vs 0.6%; *P* < .001). Mortality rates were higher for hospitalized versus nonhospitalized CDI+ patients.

**Conclusions:**

Prevention of CDI among adults 18–64 years old may significantly reduce costs and mortality.

## Introduction

*Clostridioides difficile* infection (CDI) is caused by a gram-positive, spore-forming bacterium found in soil and certain animals, including humans.^1^ Symptoms range from mild-to-moderate diarrhea to severe presentations (ie, pseudomembranous colitis, toxic megacolon) or even death.^2–4^ Risk factors include older age and healthcare exposures (eg, acute infections, antibiotic use, recent hospitalization).^5–7^ CDI complications (eg, dehydration, diarrhea, sepsis) may necessitate hospitalization, with hospitalized patients potentially transitioning to long-term care facilities and remaining at high risk for CDI recurrence or readmission.^8–10^ CDI recurrence rates range between 20% and 30%,^5,11,12^ increasing healthcare burden and costs associated with hospital readmission/duration and death due to increasing severity of subsequent infections.^8^

US CDI burden in 2019 was 58.3 and 139.1 per 100,000 person-years in 18–44- and 45–64-year-olds, respectively^13^; among ≥65-year-olds, the annual CDI incidence rate was 385.8 per 100,000 person-years.^13^ In a study using Merative MarketScan commercial and multistate Medicare/Medicaid databases, healthcare-associated (HCA) CDI rates decreased between 2011 and 2017 in 25–64-year-olds and ≥65-year-olds, whereas community-associated (CA) CDI rates increased in 25–64-year-olds (commercial insurance) and ≥65-year-olds (Medicare).^14^ Similar trends were observed from 2012 to 2019 using Optum Medicare Advantage data from ≥65-year-olds when the overall percentage of HCA CDI declined from 53.2% to 47.2% and CA CDI increased from 46.8% to 52.8%.^15^ Within this dataset, estimated 2018 mean CDI-associated healthcare costs among ≥65-year-olds were $13,500 per person within 2 months of follow-up.^15^ Excess healthcare costs were higher for hospitalized versus nonhospitalized patients with either HCA or CA CDI.^15^ CDI-associated mortality rates were up to 7.9% among elderly patients after 12 months.^15^

CDI burden and associated healthcare costs are well studied among the elderly^15^; however, data among <65-year-olds remain limited, despite previous studies suggesting a substantial burden among this age group.^16–18^ This study expands upon existing literature by providing annual CDI incidence, along with estimated healthcare and patients’ out-of-pocket costs, and associated mortality rates among adults <65-year-olds using a commercially insured claims database.

## Methods

### Study design and data source

This retrospective cohort study included US adults 18–64 years old insured under commercial plans from the Optum® Clinformatics® Data Mart, which comprises members of a large national managed care company spanning all 50 states. The database includes pharmacy- and provider-submitted claims regarding approximately 12–14 million individuals annually and >65 million unique individuals between 2000 and 2020; all submitted claims are verified and de-identified before inclusion in Optum. Claims data include standard pricing for medical, pharmacy, and inpatient charges.

### Patient consent statement

No patient consent was obtained because this study was exempt from requirements for human subjects research owing to the use of only de-identified data.

### Inclusion criteria and CDI case definition

Analyses of annual CDI incidence and outcomes were conducted in 2 separate cohorts. For assessment of annual incidence, individuals were 18–64 years old, alive, and enrolled in an Optum commercial plan on January 1 of the corresponding calendar year between 2015 and 2019. Inclusion required continuous enrollment from January 1 of the calendar year until death, disenrollment, or end of the calendar year (whichever occurred first).

The primary definition of CDI included any of the following: an inpatient claim with the *International Classification of Diseases, 9th Revision (ICD-9)* diagnosis code 008.45 or *10th Revision (ICD-10)* diagnosis code A04.7x; an outpatient claim coded for CDI plus antibiotic therapy (nontopical metronidazole, oral vancomycin, or fidaxomicin) within ±14 days of diagnosis; or an outpatient *C. difficile* toxin test (Current Procedural Terminology [CPT] codes 87230, 87324, 87493) and antibiotic therapy within ±14 days of the test. CDI cases were required to have no prior CDI (as defined above) within ≤60 days of the CDI index date, the latter based on the Centers for Disease Control and Prevention surveillance definition of new incident CDI.^19^ Because test results are not available from outpatient claims data, CDI cases were defined according to receiving the test regardless of results. Antibiotic therapy receipt indicated that the therapy was dispensed according to pharmacy claims data.

For excess costs and mortality analyses, data included claims from 2015 to 2019, and individuals were required to be 18–64 years old on the index date selected between 2016 and 2018. The index date was assigned as the first CDI diagnosis (defined above) for patients with CDI and was a randomly assigned date between 2016 and 2018 for individuals without CDI. Outcomes were evaluated for CDI+ and 1:1 propensity score-matched CDI− controls who were continuously enrolled in the database for ≥12 months before the index date and had completed 12 months of continuous enrollment after the index date, unless preceded by death.

CDI cases were classified as HCA or CA acquisition according to established guidelines.^7,15^ HCA CDI included hospital-onset CDI diagnosed during a hospitalization or other healthcare facility stay, with an index date >3 days after admission. HCA CDI also included CDI following an inpatient, skilled nursing facility, hospice, long-term care facility, or nursing home stay with >1 day duration in the 4 weeks before the CDI index date. CA CDI included outpatient onset and inpatient onset within ≤3 days of admission and with no healthcare facility overnight stay in the 12 weeks before the CDI index date. Indeterminate cases were those not meeting definitions of CA or HCA CDI.

### Measures and statistical analyses

Annual CDI incidence was assessed overall, by age group and by year from 2015 to 2019. CDI incidence rate was calculated as the number of CDI cases (defined above) each year between 2015 and 2019, divided by total follow-up time in person-years for the eligible study population in each index calendar year. Incidence rates are presented as the number of episodes per 100,000 person-years.

For outcomes analyses, healthcare utilization, costs, and mortality were compared between CDI+ and CDI− individuals. Propensity score matching (PSM) was performed using multivariable logistic regression with 61 variables, including pre-index comorbidities and healthcare utilization (Figures S1 and S2) and 1:1 matching of CDI+ and CDI– individuals as well as exact matching by 5**–**10-year age groups and index date 2-week windows. Greedy nearest-neighbor matching and a 0.1 caliper width of the SD of the logit of the propensity score were used for PSM of CDI+ and CDI− individuals. CDI+ patients without a suitable matched control were excluded from the analysis. For outcome analyses stratified by acquisition status, indeterminate case counts were low and were therefore included among HCA cases because indeterminate cases had prior healthcare facility encounters within 4–12 weeks before CDI diagnosis.

All variables, including baseline and outcome measures, were analyzed descriptively. Standardized differences were computed as absolute differences in sample means divided by the pooled SD. A standardized difference of 0.1 was used as a cutoff to indicate a clinically meaningful difference.

Healthcare costs (based on standard allowable amounts estimated by Optum) and patients’ out-of-pocket costs (eg, copays, deductibles, coinsurance) within ≤2 months of the index date were evaluated by age group, CDI acquisition type, and hospitalization status; hospitalized patients were defined as those who were hospitalized at the time of CDI diagnosis or hospitalized with a CDI diagnosis code ≤60 days post-index. Mortality was evaluated by age group and CDI hospitalization status, with proportions of CDI+ and CDI− individuals who had died at 1, 2, 3, 6, and 12 months after the index date compared using McNemar tests. Analyses were conducted using Statistical Analysis Software (SAS) version 9.4 (SAS Institute, Cary, NC, USA).

### Sensitivity analysis

To address concerns regarding the potential inclusion of false-positive cases, a sensitivity analysis was performed by excluding patients who met CDI criteria with only an outpatient *C. difficile* toxin test and antibiotic therapy within ±14 days of the test but who did not have confirmation of CDI diagnosis within 30 days. Patients included in this sensitivity analysis were only those who had either (1) an inpatient diagnosis, (2) an outpatient diagnosis plus an antibiotic prescription filled within ±14 days, or (3) a toxin test plus an antibiotic prescription filled within ±14 days, plus a subsequent CDI diagnosis within ≤30 days of the toxin test.

## Results

### Annual CDI incidence from 2015 to 2019

**Figure 1.**
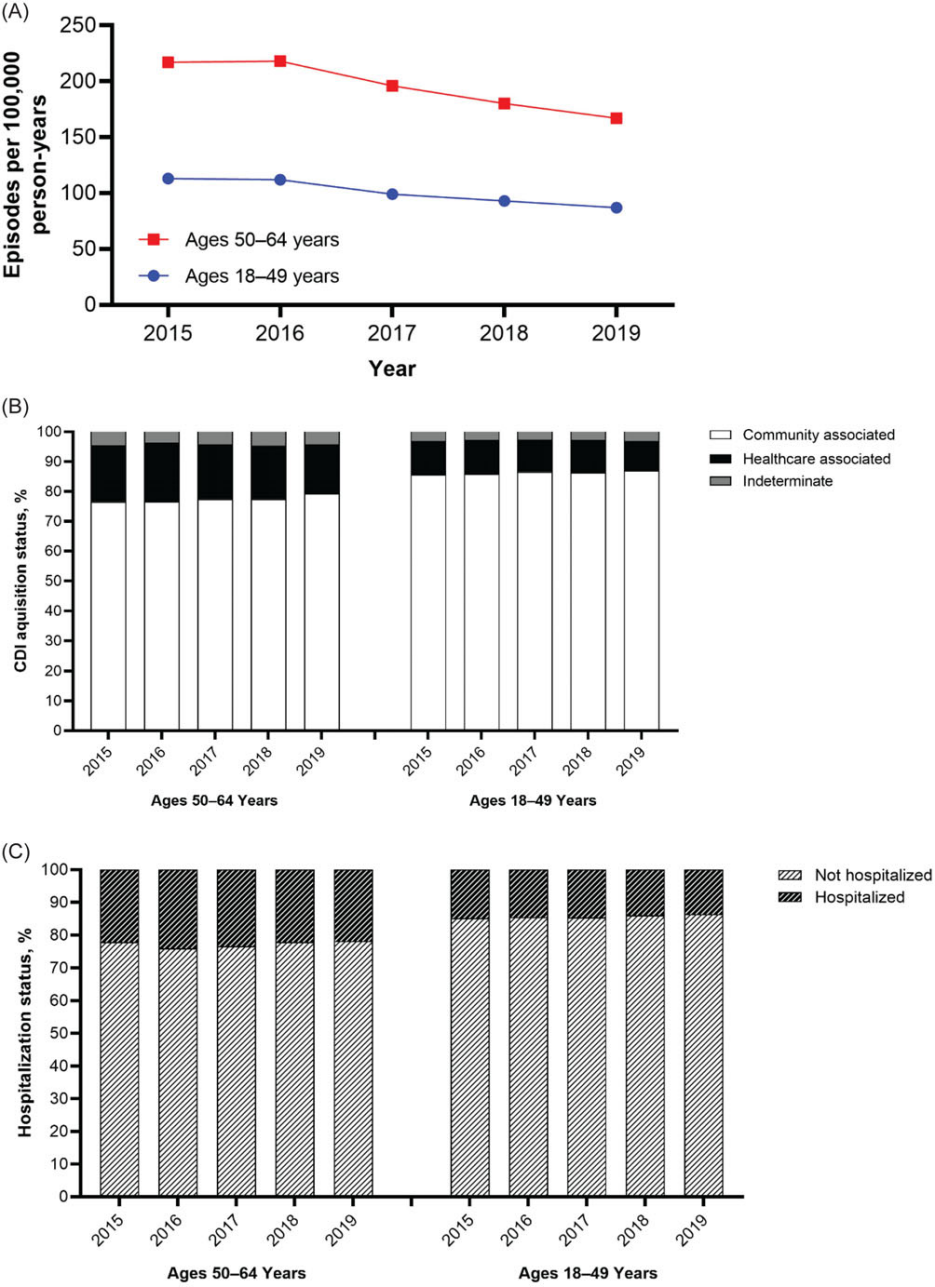
(A) Annual CDI incidence rate in each age group. (B) Percentage of CDI+ patients in each age group over time by acquisition type. (C) Percentage of CDI+ patients in each age group over time by hospitalization status. CDI, *Clostridioides difficile* infection.

Among 50–64- and 18–49-year-olds, 2015 CDI incidence was 217 and 113 cases per 100,000 person-years, respectively, which decreased by 23.0% to 167 and 87 cases per 100,000 person-years, respectively, in 2019 (Figure 1a). In both age groups, the proportion of CDI cases was higher for CA than HCA CDI, and most patients were not hospitalized (Figure 1b, 1c). Across calendar years and age groups, 10.0%–19.8% of CDI cases had HCA CDI, 4.4%–7.1% of CDI cases had indeterminate CDI, and 76.5%–86.9% of CDI cases had CA CDI (Figure 1b). Between 2015 and 2019, 21.8%–24.1% of 50–64-year-olds and 13.6%–14.9% of 18–49-year-olds with CDI were hospitalized (Figure 1c).

### Outcome results

#### Disposition and patient characteristics

Between January 2016 and December 2018, 23,513,801 adults were enrolled in the Optum database and were enrolled and alive during the year before the index year (Figure S3). Of 4,818,391 50–64-year-olds and 13,000,024 18–49-year-olds during 2016–2018, 6,787 and 7,033 patients, respectively, were CDI+ and met inclusion criteria. Patient characteristics are summarized in Table 1. Before matching, CDI+ patients were more likely to be female and have comorbidities than CDI− controls.

**Table 1.** Patient demographic and baseline clinical characteristics before propensity score matching^a^

	50–64 years age group		18–49 years age group	
**Characteristic**	**CDI+ (n = 6,787)**	**CDI– (n = 1,363,715)**	**CDI+ (n = 7,033)**	**CDI– (n = 2,663,671)**
Age, mean (SD), y	57.2 (4.2)	56.6 (4.1)	36.1 (9.2)	34.6 (9.2)
Age range, n (%), y				
18–29	n/a	n/a	1,812 (25.8)	841,300 (31.6)
30–39	n/a	n/a	2,224 (31.6)	883,265 (33.2)
40–49	n/a	n/a	2,997 (42.6)	939,106 (35.3)
50–54	1,999 (29.5)	487,531 (35.8)	n/a	n/a
55–59	2,436 (35.9)	488,854 (35.8)	n/a	n/a
60–64	2,352 (34.7)	387,330 (28.4)	n/a	n/a
Male sex, n (%)	2,782 (41.0)	687,049 (50.4)	2,886 (41.0)	1,380,603 (51.8)
US region, n (%)				
Northeast	555 (8.2)	119,267 (8.7)	677 (9.6)	247,703 (9.3)
Midwest	2,108 (31.1)	389,051 (28.5)	2,052 (29.2)	682,453 (25.6)
South	2,882 (42.5)	554,388 (40.7)	3,008 (42.8)	1,051,114 (39.5)
West	1,232 (18.2)	274,859 (20.2)	1,285 (18.3)	571,517 (21.5)
Type of first incident CDI case, n (%)				
Hospitalization or other inpatient facility	1,264 (18.6)	n/a	792 (11.3)	n/a
Outpatient	605 (8.9)	n/a	662 (9.4)	n/a
Toxin test and antibiotic	4,918 (72.5)	n/a	5,579 (79.3)	n/a
Acquisition status of first incident case, n (%)				
Healthcare associated	1,055 (15.5)	n/a	693 (9.9)	n/a
Community associated	5,393 (79.5)	n/a	6,098 (86.7)	n/a
Indeterminate	339 (5.0)	n/a	242 (3.4)	n/a
Charlson Comorbidity Index, mean (SD)	2.0 (2.6)	0.5 (1.1)	0.9 (1.7)	0.2 (0.6)
Charlson Comorbidity Index, n (%)				
0	2,748 (40.5)	972,584 (71.3)	4,452 (63.3)	2,363,815 (88.7)
1	1,337 (19.7)	220,347 (16.2)	1,344 (19.1)	224,249 (8.4)
2	859 (12.7)	100,607 (7.4)	489 (7.0)	50,343 (1.9)
3+	1,843 (27.2)	70,177 (5.1)	748 (10.6)	25,264 (0.9)

CDI, *Clostridioides difficile* infection; CDI+, CDI positive; CDI–, CDI negative; n/a, not applicable.

a Standardized differences for baseline demographic and clinical characteristics after propensity score matching are provided in Figures S1 and S2.

There were 6,332 CDI+ patients and matched CDI− controls in the 50–64-year age group, and 6,667 CDI+ patients and matched CDI− controls in the 18–49-year age group following 1:1 PSM (Figure S3). Following PSM, baseline characteristics for CDI+ cases and CDI− controls were well matched within age groups (standardized differences shown in Figure S1−S2).

#### Costs and healthcare utilization

**Figure 2.**
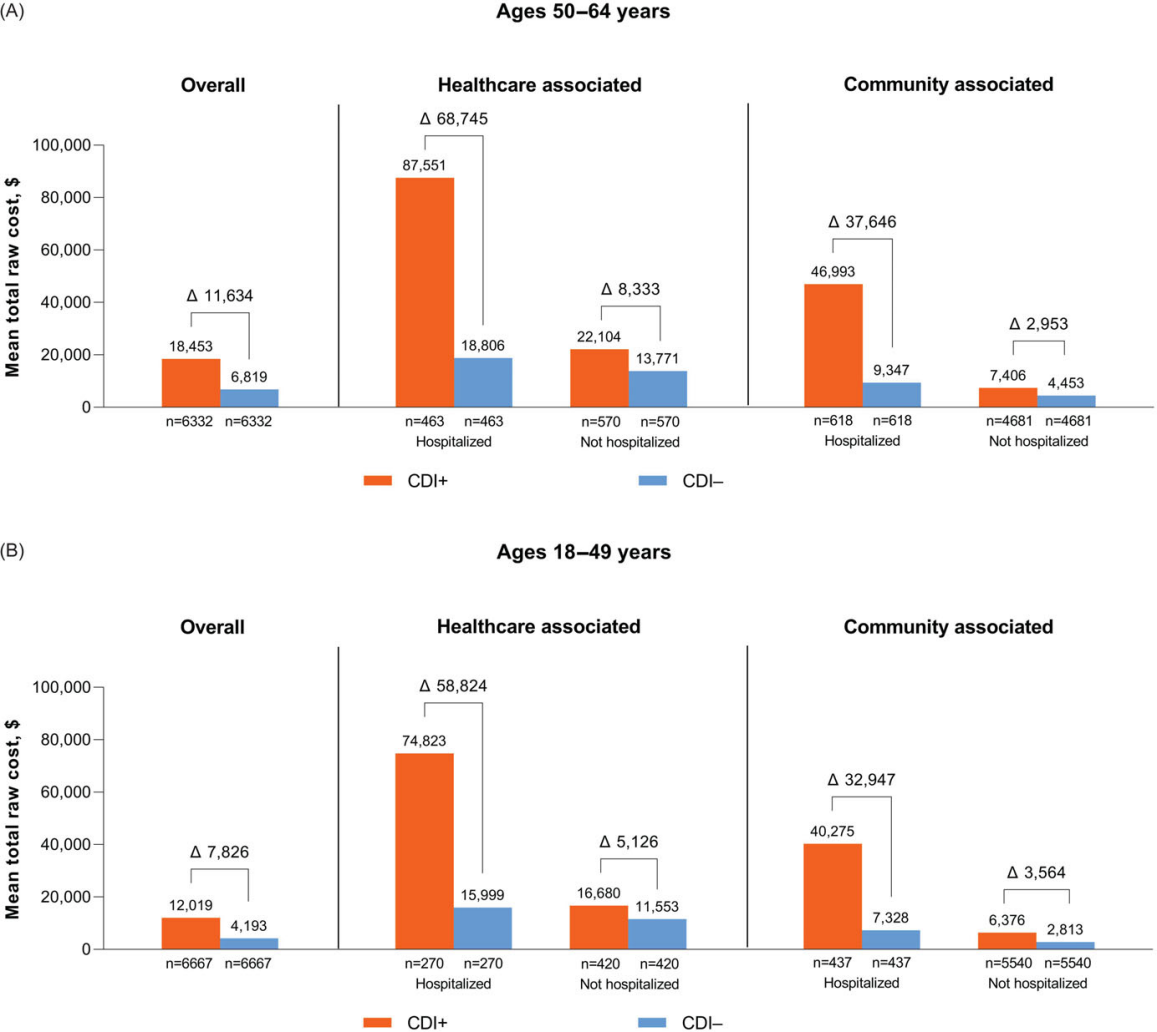
Healthcare costs at 2 months post-index by CDI acquisition type and hospitalization status for patients (A) 50–64 or (B) 18–49 years of age. Differences between CDI+ and CDI– groups are reported. Δ, difference; CDI, *Clostridioides difficile* infection; CDI+, CDI positive; CDI–, CDI negative. Costs are shown in 2019 US dollars.

Overall mean total healthcare costs at 2 months post-index were $18,453 for CDI+ and $6,819 for CDI− among 50–64-year-olds and $12,019 for CDI+ and $4,193 for CDI− among 18–49-year-olds, with differences of $11,634 and $7,826, respectively. Compared with CA CDI, HCA CDI was associated with higher total healthcare costs (Figure 2, Table S1). Overall mean out-of-pocket costs for CDI+ and CDI− patients, respectively, were $990 and $417 (difference, $573) for 50–64-year-olds and $954 and $311 (difference, $642) for 18–49-year-olds. Higher out-of-pocket costs were observed with CA versus HCA CDI in both age groups (Table S1).

Higher overall healthcare costs among CDI cases in both age groups were driven primarily by inpatient hospitalization costs, followed by outpatient costs (Table S1). Among 50–64-year-olds, mean total healthcare costs for hospitalized HCA CDI+ patients were $68,745 higher than matched CDI− controls and $37,646 higher for hospitalized patients with CA CDI (Table S1; Figure 2A). Among CDI+ nonhospitalized patients, mean total healthcare costs were $8333 and $2953 higher for patients with HCA and CA CDI, respectively, compared with CDI− controls. Mean total out-of-pocket costs for hospitalized HCA and hospitalized CA CDI+ patients were $722 and $1692 higher, respectively, than matched CDI− controls (Table S1). Among nonhospitalized CDI+ patients, mean out-of-pocket costs were $125 and $465 higher for patients with HCA and CA CDI, respectively, than for CDI− controls.

Comparable findings were observed among 18–49-year-olds, although mean costs were generally lower than for the older group (Table S1, Figure 2B). Mean total healthcare costs for hospitalized CDI+ patients were $58,824 and $32,947 higher for HCA and CA CDI, respectively, than CDI− controls. Mean total healthcare costs were $5,126 and $3,564 higher in nonhospitalized HCA and CA CDI+ patients, respectively, than CDI− controls. Mean total out-of-pocket costs for hospitalized HCA and CA CDI+ patients were $1,223 and $1,886 higher, respectively, than CDI− controls (Table S1). Among nonhospitalized CDI+ patients, mean out-of-pocket costs were $139 and $554 higher for patients with HCA and CA CDI, respectively, than CDI− controls.

Patterns of healthcare utilization based on acquisition status were similar between both age groups (Table 2; Tables S2 and S3). CDI was associated with an increased mean number of outpatient visits and a higher proportion of patients with emergency department visits across all patient groups, regardless of age, acquisition type, or hospitalization status. Similar results were observed for inpatient utilization; mean numbers of inpatient visits and days were higher among CDI+ patients versus controls, regardless of age, acquisition type, or CDI hospitalization status. Proportions of patients with outpatient prescriptions were higher among all groups of CDI+ patients versus CDI− controls (Table 2; Tables S2−S3), except for hospitalized 50–64-year-olds with HCA CDI.

**Table 2.** Healthcare resource utilization at 2 months post-index

	50–64 years age group			Aged 18–49 years age group		
	CDI+ (n = 6,332)	CDI– (n = 6,332)	Difference	CDI+ (n = 6,667)	CDI– (n = 6,667)	Difference
Any outpatient visits, n (%)^a^	6,241 (98.6)	4,868 (76.9)	21.7	6,608 (99.1)	4,344 (65.2)	34.0
Mean number of outpatient visits per patient (SD)^a^	9.5 (12.1)	5.2 (8.2)	4.3	7.6 (7.8)	3.5 (6.2)	4.0
Any ED visits, n (%)	1,466 (23.2)	347 (5.5)	17.7	1,908 (28.6)	485 (7.3)	21.3
Mean number of ED visits per patient (SD)	0.3 (0.6)	0.1 (0.3)	0.2	0.4 (0.7)	0.1 (0.4)	0.3
Inpatient hospitalization, n (%)	1,418 (22.4)	322 (5.1)	17.3	1,012 (15.2)	257 (3.9)	11.3
Mean number of inpatient days per patient (SD)	2.5 (7.4)	0.5 (3.2)	2.0	1.5 (5.7)	0.3 (2.8)	1.2
Any outpatient prescription, n (%)	6,069 (95.8)	5,332 (84.2)	11.6	6,363 (95.4)	4,555 (68.3)	27.1
Mean number of outpatient prescriptions per patient (SD)	7.9 (6.6)	6.3 (6.4)	1.6	5.8 (5.5)	3.7 (5.0)	2.1

CDI, *Clostridioides difficile* infection; CDI+, CDI positive; CDI–, CDI negative; ED, emergency department; n, number of patients.

Values presented after propensity score matching. Numbers and days spent in other inpatient facilities (skilled nursing facility, inpatient hospice facility, inpatient mental health/chemical dependence facility, or inpatient rehabilitation facility) are not shown in order to maintain patient de-identification due to small cell counts.

a Excludes ED visits.

#### Mortality

**Figure 3.**
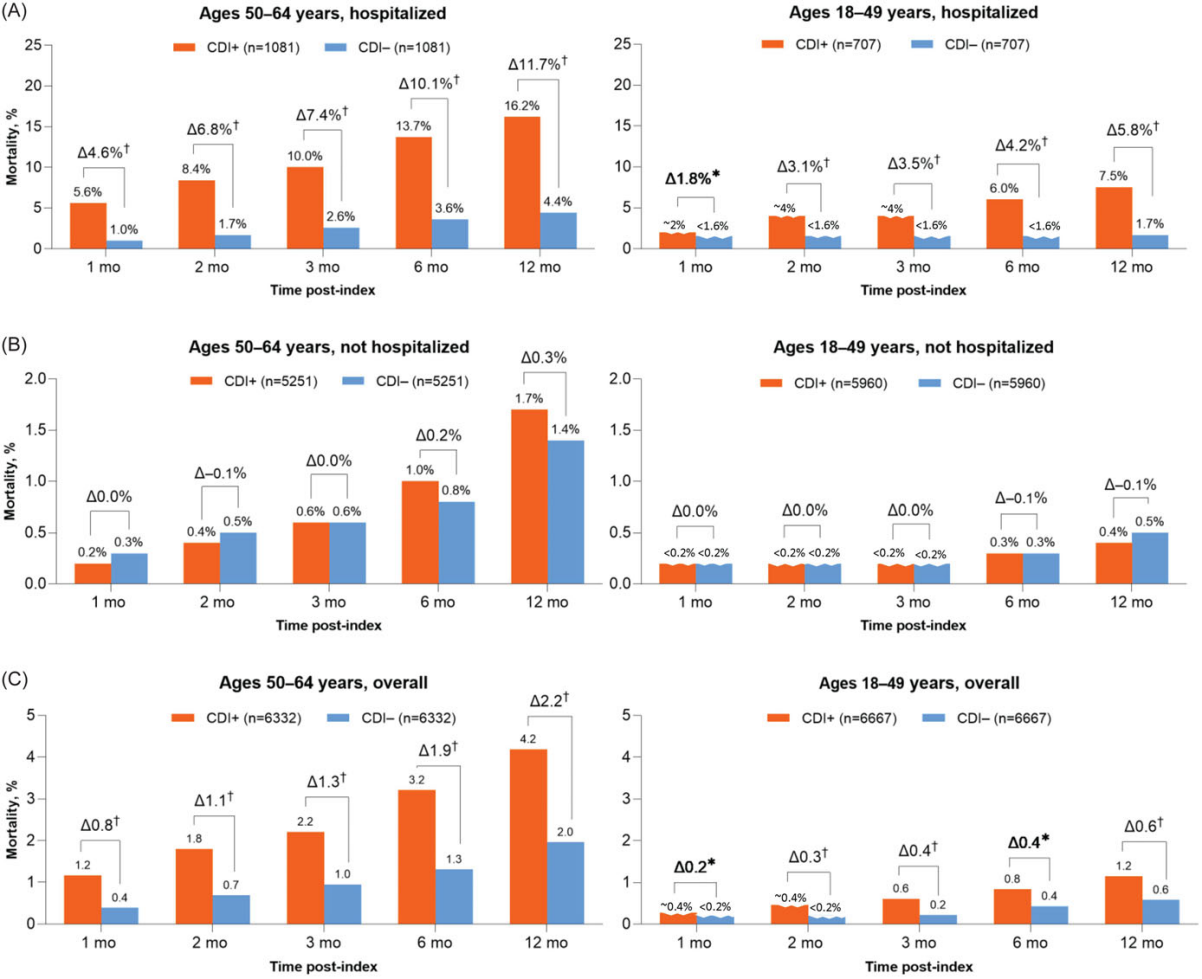
Mortality during follow-up (A, B) by CDI hospitalization status and (C) overall for patients 50–64 or 18–49 years of age. Differences between CDI+ and CDI– groups are reported. *P < .05; ^†^*P* < .001. Δ, difference; CDI, *Clostridioides difficile* infection; CDI+, CDI positive; CDI–, CDI negative. Some percentages are rounded to maintain patient de-identification.

At each follow-up between 1 and 12 months post-index, mortality rates were higher in 50–64-year-olds than 18–49-year-olds among both hospitalized and nonhospitalized patients (Figure 3). At 12 months, overall mortality among 50–64-year-olds was 4.2% for CDI+ patients versus 2.0% for CDI− controls (*P* < .001). Among 18–49-year-olds at 12 months, overall mortality was 1.2% for CDI+ patients versus 0.6% for CDI− controls (*P* < .001). Among hospitalized CDI+ patients matched to CDI– controls, excess mortality rates at 12 months post-index were 11.7% and 5.8% among the older and younger groups, respectively. In both age groups, excess mortality was higher among hospitalized versus nonhospitalized CDI+ patients and gradually increased through the 12 months after the index date (Figure 3).

#### Sensitivity analysis

Of the CDI+ patients, 2,732 (50−64-year-olds) and 2,388 (18−49-year-olds) met the stringent CDI definition requiring a CDI diagnosis. Residual imbalances were observed between matched CDI+ patients and CDI− controls for baseline comorbidities in these smaller subgroups (Tables S4 and S5). Outcomes were generally consistent with the main analysis; however, patterns of findings for healthcare utilization and costs showed higher overall out-of-pocket costs ($1,258 among 50–64-year-olds and $1,206 among 18–49-year-olds) and total number of outpatient or inpatient visits in the sensitivity analysis compared with the primary analyses (Tables S6, S7, S8).

## Discussion

This large retrospective cohort study showed that annual CDI incidence among 18–64-year-olds was similar in 2015 and 2016, gradually decreasing to 167 and 87 cases per 100,000 person-years in older and younger age groups, respectively, by 2019. Most CDI patients were not hospitalized, and most CDI cases were CA versus HCA. Incidence rates and trends regarding the proportion of cases that were CA versus HCA were consistent with those reported in a study of <65-year-old CDI+ patients within the Veterans Health Administration database.^20^

CDI was associated with increases in total healthcare costs of $11,634 in 50–64-year-olds and $7,826 in 18**–**49-year-olds. Hospitalization drove a large portion of CDI-associated cost increases for patients with both HCA and CA infections. CDI was also associated with increased out-of-pocket costs by $573 and $642 in 50–64-year-olds and 18–49-year-olds, respectively. Compared with HCA CDI, CA CDI was associated with higher out-of-pocket costs in both age groups, regardless of CDI hospitalization status. Healthcare utilization (eg, mean number of outpatient, emergency department, inpatient visits) was higher in the CDI+ than CDI− group, regardless of age, acquisition type, or CDI hospitalization status. The only exception to this trend was for the proportion of patients with outpatient prescriptions among hospitalized 50–64-year-olds with HCA CDI, which was likely because hospitalized patients have more limited opportunities for filling outpatient prescriptions.

Differences in overall mortality between the CDI+ and CDI− groups increased through 12 months after the index date and were higher among hospitalized versus nonhospitalized CDI+ patients. Within 12 months, CDI was associated with 2.2% and 0.6% excess mortality among 50–64-year-olds and 18–49-year-olds, respectively. Among hospitalized CDI+ patients, respective excess mortality in the older and younger age groups reached 11.7% and 5.8%, respectively; however, this may be overestimated because the matched CDI– controls were not necessarily hospitalized, particularly those who were matched with HCA hospitalized CDI+ cases.

In a similar analysis of US claims data from 2010 to 2014, Zhang and colleagues reported mean excess 6-month costs of $26,663 and $21,160 for primary (nonrecurrent) CDI among <65-year-olds and ≥65-year-olds, respectively.^11^ Using the MarketScan commercial database for adults 25**–**64 years of age, Sahrmann and colleagues calculated that 1-year CDI-excess costs for HCA CDI and CA CDI were $43,127 and $13,105, respectively.^18^ A Canadian population study using a PSM cohort further reported a 1-year 13% mortality risk due to community-onset CDI among all-aged individuals.^21^ Using Medicare claims data for ≥66-year-olds, Olsen and colleagues reported a similar CDI-excess mortality risk of 10.9% at 1-year follow-up.^22^ Our results are consistent with these findings in both young adult and elderly populations and highlight the vulnerability of younger hospitalized adults with CDI, although they are at relatively lower mortality risk than older patients.

Given the considerable burden of CDI among <65-year-olds and the paucity of available data, further research is needed to determine rates of recurrence, specific morbidities, and high-risk groups within younger US adults. We have previously reported that CDI is associated with septicemia and urinary tract infections among Medicaid enrollees 25–64 years old^16^; additional studies should be conducted to characterize the prevalence of complications such as colitis and irritable bowel syndrome.

Strengths of this analysis include characterization of incidence, healthcare utilization, costs, and mortality associated with CDI among US adults <65 years old, for whom existing data are limited. However, there were some important limitations. Retrospective observational studies could lead to bias owing to unmeasured confounding variables. Moreover, claims databases may be associated with underreporting or misclassification of health outcomes,^23,24^ and the only information available regarding mortality is the month and date of death, without details of the associated cause. Because Optum includes only members covered under commercial healthcare plans, results may not fully represent the 18**–**64-year-old population. The cost analysis was limited to costs incurred ≤2 months after diagnosis. Indeterminate CDI cases were considered HCA for the costs and mortality analyses, given the small numbers of such cases. Furthermore, CDI diagnosis codes may lack the specificity required to determine whether an event of interest occurred, based on a meta-analysis of 7 studies in which positive predictive value was only 72% for the CDI ICD-9 diagnosis code.^25^ The use of laboratory data in this study may help minimize under-reporting of CDI; however, this may also lead to misclassification without the availability of diagnostic testing results. Finally, it is important to note that CDI+ patients and CDI– controls were matched on propensity score where prior hospitalization status (≤90 days prior to index date) but not hospitalization status post-index was included in the propensity score model. Thus, where hospitalized CDI+ patients were compared with CDI– controls in the analysis stratified by hospitalization status (ie, hospitalization status at the time of diagnosis or ≤60 days post-index), most CDI– controls were not hospitalized. This methodology could have resulted in an overestimation of CDI-excess costs and mortality in the hospitalized group.

Among 18–49- and 50–64-year-olds, CDI was associated with substantially higher healthcare costs and mortality compared with matched CDI− controls. Identification and prevention of CDI among younger adults who are at increased risk for infection have the potential to significantly reduce both healthcare system and patient costs and mortality.

## Supporting information

Yu et al. supplementary material Yu et al. supplementary material

## Data Availability

Upon request, and subject to review, Pfizer will provide the data that support the findings of this study. Subject to certain criteria, conditions, and exceptions, Pfizer may also provide access to the related individual de-identified participant data. See https://www.pfizer.com/science/clinical-trials/trial-data-and-results for more information.

## Supplementary material

For supplementary material accompanying this paper visit http://doi.org/10.1017/ash.2024.400. click here to view supplementary material
