# Supplementary material for: Incidence, healthcare and out-of-pocket costs, and mortality of Clostridioides difficile infection among US adults aged 18 to 64 years
Source: Antimicrob Steward Healthc Epidemiol. 2024 Dec 11;4(1):e215. doi: 10.1017/ash.2024.400 (PMC11696600; doi:10.1017/ash.2024.400)
Supplement: Yu et al. supplementary material [file S2732494X24004005sup001.pdf]

Supplementary Appendix

Figure S1. Absolute Standardized Differences of Variables Included in the Propensity Score Before and After Matching in the 50–64 Years Age Group

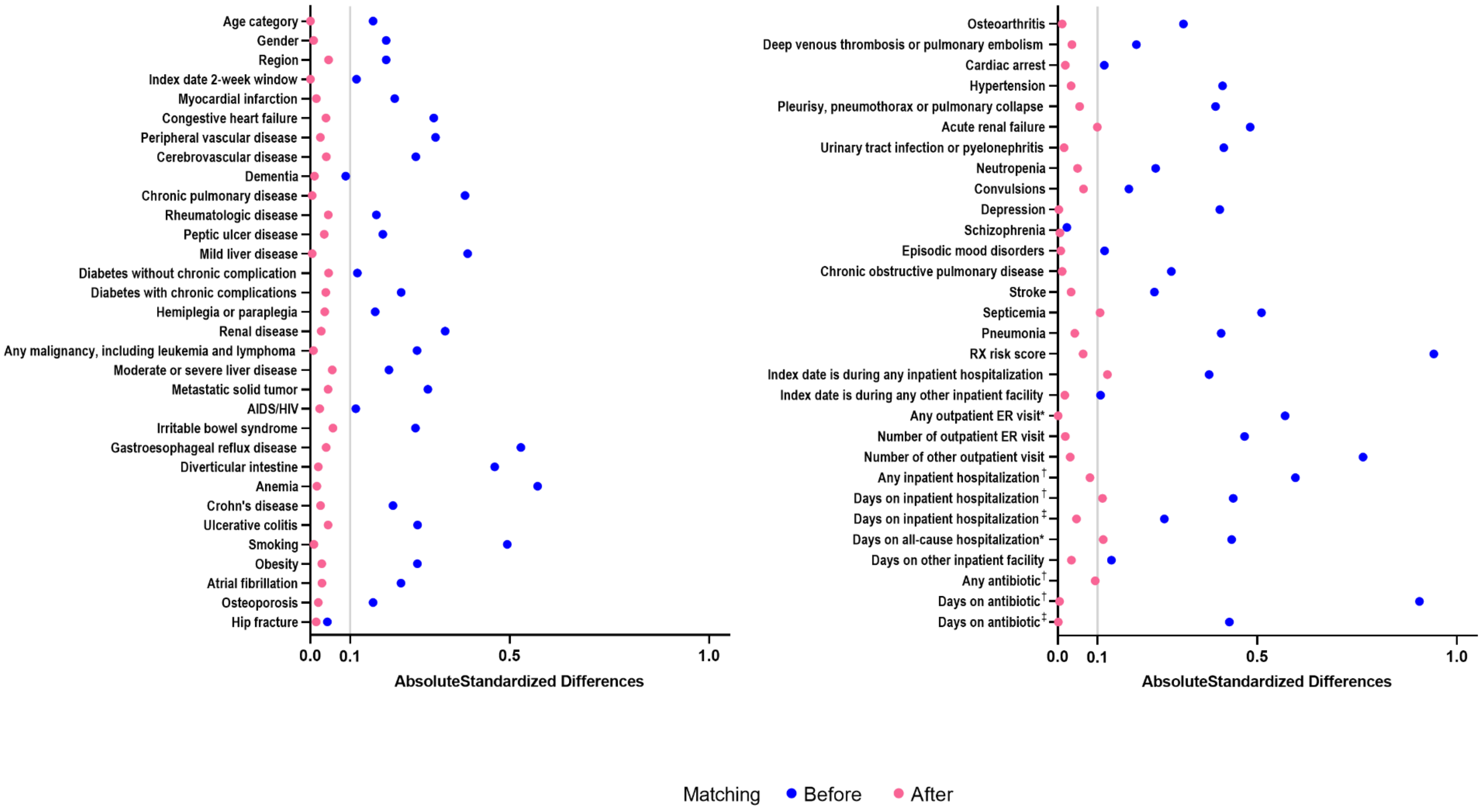

ER, emergency room.
Standardized differences ≤0.1 signify acceptable balance. Conditions and healthcare resource utilization are during 1-year pre-index unless otherwise specified.
*2 months pre-index
†90 days pre-index
‡91 days to 12 months pre-index

Figure S2. Absolute Standardized Differences of Variables Included in the Propensity Score Before and After Matching in the 18–49 Years Age Group.

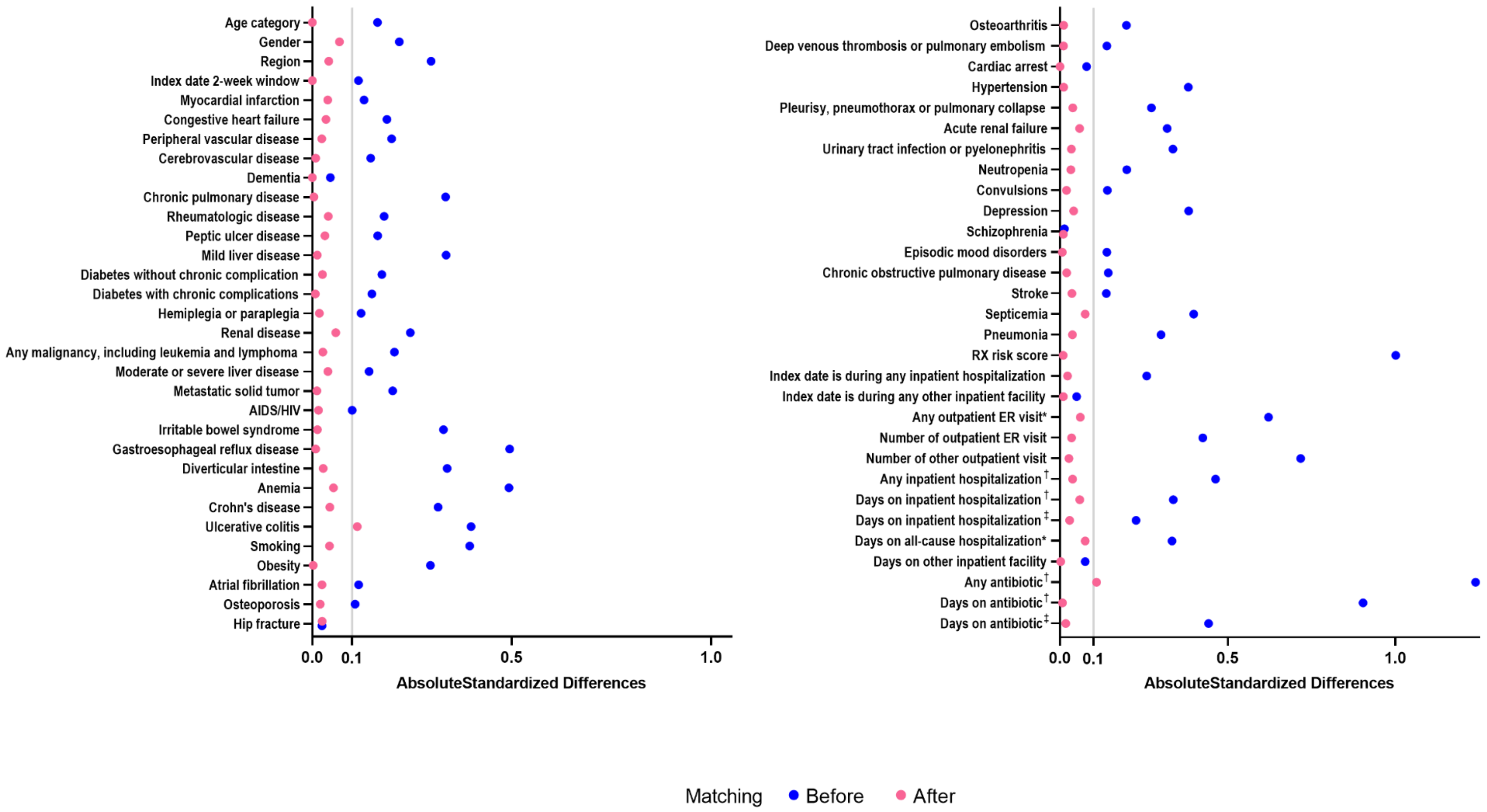

ER, emergency room.

Standardized differences ≤0.1 signify acceptable balance. Conditions and healthcare resource utilization are during 1-year pre-index unless otherwise specified.

*2 months pre-index

†90 days pre-index

‡91 days to 12 months pre-index

Figure S3. Outcomes Analysis Cohorts

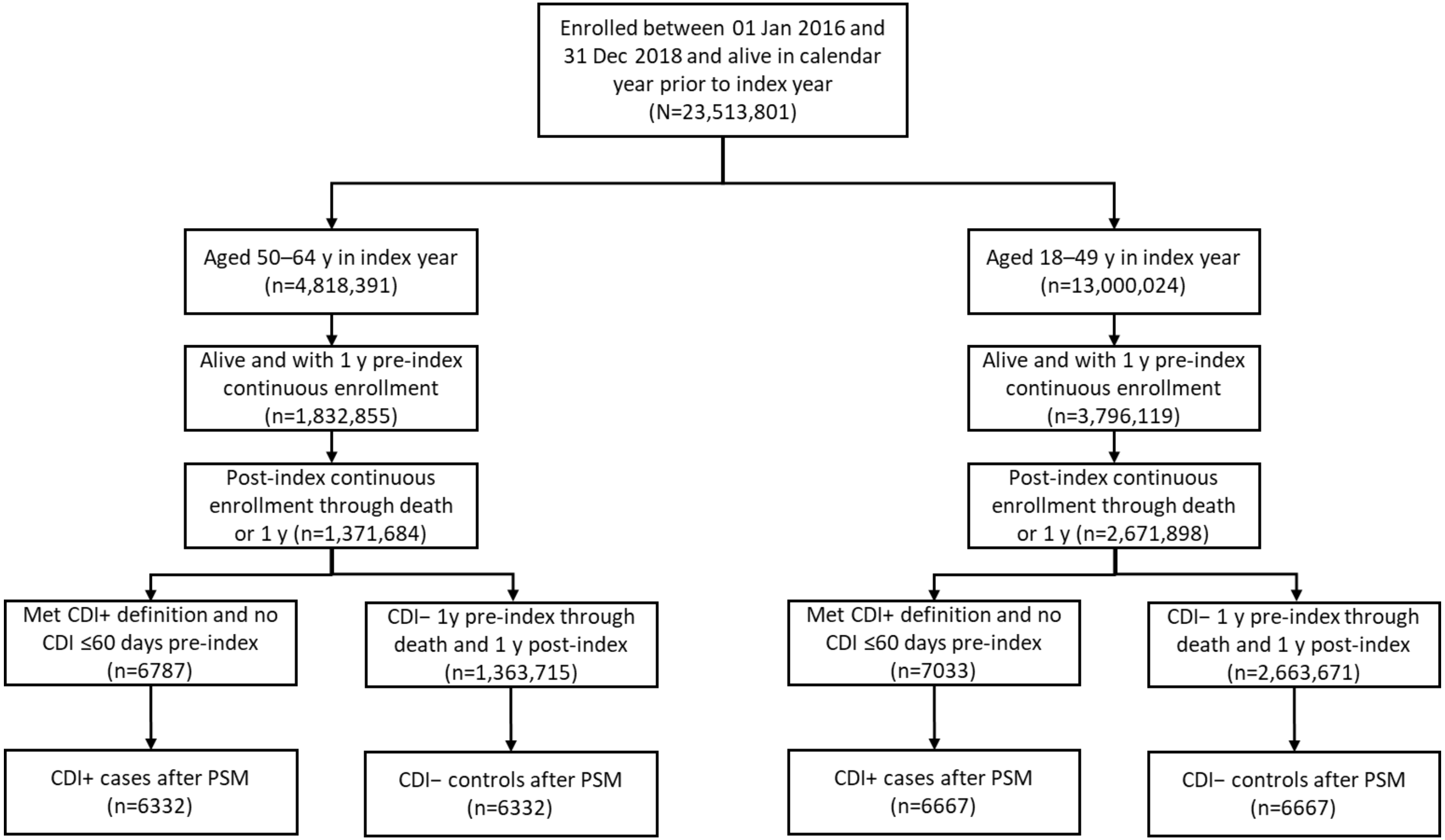

CDI, *Clostridioides difficile* infection; CDI+, CDI positive; CDI–, CDI negative; PSM, propensity score matching.

Table S1. Healthcare and Patient Out-of-Pocket Costs at 2 Months Post-Index by CDI Acquisition, Hospitalization Status, and Age Group

	Healthcare Associated, $						Community Associated, $								
	Hospitalized[a]			Not Hospitalized[a]			Hospitalized[a]			Not Hospitalized[a]			Overall, $		
Age Group	CDI+	CDI–	Difference	CDI+	CDI–	Difference	CDI+	CDI–	Difference	CDI+	CDI–	Difference	CDI+	CDI–	Difference
50–64 y	n=463	n=463		n=570	n=570		n=618	n=618		n=4681	n=4681		n=6332	n=6332	
Outpatient[b]	13,815 (30,232)	7972 (16,980)	5842	11,071 (21,043)	5997 (18,349)	5074	7746 (19,711)	5333 (14,276)	2413	4448 (12,257)	2329 (7687)	2119	6051 (16,256)	3365 (10,872)	2686
Emergency department	537 (2384)	173 (749)	364	481 (1161)	165 (789)	316	557 (2703)	319 (4386)	238	442 (1383)	95 (1246)	347	464 (1632)	129 (1768)	335
Inpatient hospitalization	67,740 (247,794)	8193 (30,719)	59,548	7862 (29,867)	5921 (26,951)	1941	35,741 (44,674)	2356 (10,332)	33,385	1190 (7961)	1148 (11,194)	42	10,029 (71,893)	2211 (15,552)	7819
Other inpatient facility[c]	3240 (14,780)	675 (4748)	2566	917 (4882)	159 (1524)	758	1327 (6724)	100 (1136)	1227	40 (1496)	26 (975)	14	479 (4993)	93 (1646)	386
Outpatient prescription	2218 (5217)	1793 (4260)	425	1774 (4231)	1530 (4379)	244	1621 (3325)	1239 (3537)	382	1286 (3426)	855 (3439)	431	1431 (3664)	1022 (3620)	409
Total healthcare	87,551 (265,353)	18,806 (38,947)	68,745	22,104 (41,032)	13,771 (33,809)	8333	46,993 (51,233)	9347 (21,329)	37,646	7406 (16,522)	4453 (15,122)	2953	18,453 (79,166)	6819 (21,128)	11,634
Total out-of-pocket costs[d]	1366 (1979)	644 (1429)	722	639 (1491)	513 (940)	125	2178 (2227)	486 (879)	1692	839 (1173)	374 (803)	465	990 (1470)	417 (887)	573
18–49 y	n=270	n=270		n=420	n=420		n=437	n=437		n=5540	n=5540		n=6667	n=6667	
Outpatient[b]	12,184 (22,798)	8062 (24,116)	4121	7865 (13,978)	5094 (13,749)	2771	7116 (19,099)	3252 (9339)	3865	3732 (8089)	1486 (5893)	2246	4557 (10,748)	2095 (8504)	2461
Emergency department	535 (1388)	255 (865)	280	663 (1643)	302 (1003)	361	539 (1198)	335 (3296)	204	561 (1572)	130 (943)	431	565 (1547)	159 (1244)	406
Inpatient hospitalization	59,380 (76,409)	5876 (31,255)	53,503	5995 (25,066)	4721 (30,318)	1274	30,817 (37,293)	2826 (17,061)	27,991	935 (6820)	724 (6333)	211	5579 (24,117)	1322 (12,312)	4257
Other inpatient facility[c]	915 (5886)	183 (2865)	732	190 (1612)	79 (971)	111	92 (1114)	25 (515)	68	0 (0)	1 (76)	−1	55 (1295)	15 (644)	40
Outpatient prescription	1809 (3725)	1622 (5449)	187	1967 (9050)	1358 (4109)	609	1711 (3606)	891 (3332)	820	1148 (5048)	472 (2321)	676	1263 (5273)	602 (2750)	662
Total healthcare	74,823 (80,522)	15,999 (43,565)	58,824	16,680 (33,378)	11,553 (34,056)	5126	40,275 (43,859)	7328 (21,993)	32,947	6376 (13,092)	2813 (9733)	3564	12,019 (28,980)	4193 (16,466)	7826
Total out-of-pocket costs[d]	1901 (4102)	678 (1617)	1223	593 (1142)	454 (944)	139	2318 (2268)	432 (853)	1886	827 (1251)	273 (713)	554	954 (1605)	311 (800)	642

CDI, *Clostridioides difficile* infection; CDI+, CDI positive; CDI–, CDI negative.

Costs are presented in US dollars after propensity score matching. Costs were adjusted to 2019 US dollars using the medical care component of the Consumer Price Index.

Costs within CDI groups are displayed as mean (SD).

[a]Descriptions of "hospitalized" and "not hospitalized" refer to the CDI+ patients only; matched controls were not necessarily hospitalized. Hospitalized patients were defined as those who were hospitalized at time of CDI diagnosis or hospitalized with a CDI diagnosis code within 60 days post-index.

[b]Excludes emergency department visits.

[c]Skilled nursing facility, inpatient hospice facility, inpatient mental health/chemical dependence facility, or inpatient rehabilitation facility.

[d]Out-of-pocket costs encompassed copays, deductibles, and coinsurance payments.

Table S2. Healthcare Resource Utilization at 2 Months Post-Index by CDI Acquisition and Hospitalization Status in the 50–64 Years Age Group

	Healthcare Associated						Community Associated					
	Hospitalized[a]			Not Hospitalized[a]			Hospitalized[a]			Not Hospitalized[a]		
	CDI+ (n=463)	CDI– (n=463)	Difference	CDI+ (n=570)	CDI– (n=570)	Difference	CDI+ (n=618)	CDI– (n=618)	Difference	CDI+ (n=4681)	CDI– (n=4681)	Difference
Any outpatient visits, n (%)[b]	419 (90.5)	422 (91.1)	–0.6	567 (99.5)	497 (87.2)	12.3	574 (92.9)	513 (83.0)	9.9	4681 (100.0)	3436 (73.4)	26.6
Mean number of outpatient visits per patient (SD)[b]	17.8 (23.7)	9.8 (11.9)	8.0	15.6 (15.7)	8.0 (10.5)	7.6	10.5 (14.3)	6.8 (10.7)	3.7	7.9 (8.5)	4.2 (6.6)	3.7
Any ED visits, n (%)	80 (17.3)	42 (9.1)	8.2	156 (27.4)	49 (8.6)	18.8	117 (18.9)	44 (7.1)	11.8	1113 (23.8)	212 (4.5)	19.2
Mean number of ED visits per patient (SD)	0.3 (0.9)	0.1 (0.4)	0.2	0.3 (0.6)	0.1 (0.3)	0.2	0.3 (0.7)	0.1 (0.5)	0.2	0.3 (0.6)	0.1 (0.3)	0.2
Inpatient hospitalization, n (%)	463 (100.0)	78 (16.8)	83.2	97 (17.0)	71 (12.5)	4.6	618 (100.0)	48 (7.8)	92.2	240 (5.1)	125 (2.7)	2.5
Mean number of inpatient days per patient (SD)	15.4 (14.7)	1.8 (6.4)	13.6	1.6 (5.3)	1.2 (4.9)	0.4	9.7 (10.8)	0.6 (2.6)	9.1	0.3 (2.2)	0.2 (2.4)	0.1
Any outpatient prescription, n (%)	391 (84.4)	425 (91.8)	–7.3	554 (97.2)	517 (90.7)	6.5	571 (92.4)	547 (88.5)	3.9	4553 (97.3)	3843 (82.1)	15.2
Mean number of outpatient prescriptions per patient (SD)	9.9 (8.5)	9.3 (7.6)	0.6	9.6 (7.7)	8.8 (7.8)	0.8	9.0 (7.2)	7.5 (6.8)	1.5	7.3 (6.0)	5.5 (5.9)	1.8

CDI, *Clostridioides difficile* infection; CDI+, CDI positive; CDI–, CDI negative; ED, emergency department; n, number of patients. Values presented after propensity score matching. Numbers and days spent in other inpatient facilities (skilled nursing facility, inpatient hospice facility, inpatient mental health/chemical dependence facility, or inpatient rehabilitation facility) are not shown in order to maintain patient de-identification due to small cell counts.

[a]Descriptions of “hospitalized” and “not hospitalized” refer to the CDI+ patients only; matched controls were not necessarily hospitalized. Hospitalized patients were defined as those who were hospitalized at time of CDI diagnosis or hospitalized with a CDI diagnosis code within 60 days post-index.

[b]Excludes ED visits.

Table S3. Healthcare Resource Utilization at 2 Months Post-Index by CDI Acquisition and Hospitalization Status in the 18–49 Years Age Group

	Healthcare Associated						Community Associated					
	Hospitalized[a]			Not Hospitalized[a]			Hospitalized[a]			Not Hospitalized[a]		
	CDI+ (n=270)	CDI– (n=270)	Difference	CDI+ (n=420)	CDI– (n=420)	Difference	CDI+ (n=437)	CDI– (n=437)	Difference	CDI+ (n=5540)	CDI– (n=5540)	Difference
Any outpatient visits, n (%)[b]	238 (88.1)	225 (83.3)	4.8	420 (100.0)	354 (84.3)	15.7	410 (93.8)	328 (75.1)	18.8	5540 (100.0)	3437 (62.0)	38.0
Mean number of outpatient visits per patient (SD)[b]	12.7 (12.7)	7.8 (10.8)	4.9	11.7 (13.0)	6.3 (7.7)	5.4	9.4 (10.2)	4.9 (7.4)	4.6	6.8 (6.4)	3.0 (5.4)	3.8
Any ED visits, n (%)	62 (23.0)	33 (12.2)	10.7	125 (29.8)	61 (14.5)	15.2	116 (26.5)	39 (8.9)	17.6	1605 (29.0)	352 (6.4)	22.6
Mean number of ED visits per patient (SD)	0.4 (0.9)	0.2 (0.6)	0.2	0.4 (0.8)	0.2 (0.6)	0.2	0.4 (0.7)	0.1 (0.6)	0.2	0.4 (0.6)	0.1 (0.4)	0.3
Inpatient hospitalization, n (%)	270 (100.0)	31 (11.5)	88.5	62 (14.8)	44 (10.5)	4.3	436 (99.8)	35 (8.0)	91.8	244 (4.4)	147 (2.7)	1.8
Mean number of inpatient days per patient (SD)	16.4 (16.3)	1.6 (7.0)	14.8	1.4 (5.0)	1.2 (5.9)	0.2	8.1 (8.5)	0.7 (3.3)	7.4	0.3 (1.9)	0.2 (1.9)	0.1
Any outpatient prescription, n (%)	237 (87.8)	221 (81.9)	5.9	406 (96.7)	355 (84.5)	12.1	410 (93.8)	345 (78.9)	14.9	5310 (95.8)	3634 (65.6)	30.3
Mean number of outpatient prescriptions per patient (SD)	9.5 (8.6)	6.6 (7.4)	2.9	7.6 (6.9)	6.2 (6.9)	1.4	8.2 (6.4)	4.8 (5.5)	3.4	5.3 (4.9)	3.3 (4.5)	2.1

CDI, *Clostridioides difficile* infection; CDI+, CDI positive; CDI–, CDI negative; ED, emergency department; n, number of patients. Values presented after propensity score matching. Numbers and days spent in other inpatient facilities (skilled nursing facility, inpatient hospice facility, inpatient mental health/chemical dependence facility, or inpatient rehabilitation facility) are not shown in order to maintain patient de-identification due to small cell counts.

[a]Descriptions of “hospitalized” and “not hospitalized” refer to the CDI+ patients only; matched controls were not necessarily hospitalized. Hospitalized patients were defined as those who were hospitalized at time of CDI diagnosis or hospitalized with a CDI diagnosis code within 60 days post-index.
[b]Excludes ED visits.

Table S4. Patient Demographic and Baseline Clinical Characteristics Before and After Propensity Score Matching in the 50–64 Years Age Group (Sensitivity Analysis Requiring a CDI Diagnosis[a])

	Before Propensity Score Matching			After Propensity Score Matching		
**Characteristic**	**CDI+ (n=2732)**	**CDI− (n=1,363,715)**	**Std Diff**	**CDI+ (n=2368)**	**CDI− (n=2368)**	**Std Diff**
Age, mean (SD), y	57.4 (4.3)	56.6 (4.1)	0.21[b]	57.5 (4.2)	57.4 (4.1)	0.01
Age range, n (%), y			0.21[b]			0.00
50–54	788 (28.8)	487,531 (35.8)		673 (28.4)	673 (28.4)	
55–59	914 (33.5)	488,854 (35.8)		805 (34.0)	805 (34.0)	
60–64	1030 (37.7)	387,330 (28.4)		890 (37.6)	890 (37.6)	
Male sex, n (%)	1138 (41.7)	687,049 (50.4)	0.18[b]	967 (40.8)	920 (38.9)	0.04
US region, n (%)			0.20[b]			0.11[b]
Northeast	215 (7.9)	119,267 (8.7)		184 (7.8)	210 (8.9)	
Midwest	921 (33.7)	389,051 (28.5)		802 (33.9)	747 (31.5)	
South	1049 (38.4)	554,388 (40.7)		906 (38.3)	991 (41.8)	
West	543 (19.9)	274,859 (20.2)		472 (19.9)	420 (17.7)	
Type of first incident CDI case, n (%)			n/a			n/a
Hospitalization or other inpatient facility	1264 (46.3)	n/a		969 (40.9)	n/a	
Outpatient	605 (22.1)	n/a		565 (23.9)	n/a	
Toxin test and antibiotic	863 (31.6)	n/a		834 (35.2)	n/a	
Acquisition status of first incident case, n (%)			n/a			n/a
Healthcare associated	800 (29.3)	n/a		530 (22.4)	n/a	
Community associated	1747 (63.9)	n/a		1689 (71.3)	n/a	
Indeterminate	185 (6.8)	n/a		149 (6.3)	n/a	
Charlson Comorbidity Index, mean (SD)	2.9 (3.1)	0.5 (1.1)	1.00[b]	2.5 (2.9)	1.9 (2.4)	0.22[b]

Charlson Comorbidity Index, n (%)			1.12[b]			0.20[b]
0	786 (28.8)	972,584 (71.3)		764 (32.3)	896 (37.8)	
1	469 (17.2)	220,347 (16.2)		442 (18.7)	482 (20.4)	
2	342 (12.5)	100,607 (7.4)		312 (13.2)	352 (14.9)	
3+	1135 (41.5)	70,177 (5.1)		850 (35.9)	638 (26.9)	

CDI, *Clostridioides difficile* infection; CDI+, CDI positive; CDI–, CDI negative; n/a, not applicable; Std diff, standardized difference.

[a]Sensitivity analysis was performed on outcomes by excluding those patients without a CDI diagnosis. Included patients met criteria for either 1) inpatient CDI diagnosis, 2) an antibiotic prescription filled within 2 weeks of an outpatient CDI diagnosis, or 3) an antibiotic prescription filled within 2 weeks of a toxin test, plus a subsequent CDI diagnosis within 30 days.

[b]Absolute values >0.1 considered significant.

Table S5. Patient Demographic and Baseline Clinical Characteristics Before and After Propensity Score Matching in the 18–49 Years Age Group (Sensitivity Analysis Requiring a CDI Diagnosis[a])

	Before Propensity Score Matching			After Propensity Score Matching		
**Characteristic**	**CDI+ (n=2388)**	**CDI– (n=2,663,671)**	**Std Diff**	**CDI+ (n=2134)**	**CDI– (n=2134)**	**Std Diff**
Age, mean (SD), y	36.2 (9.3)	34.6 (9.2)	0.18[b]	36.4 (9.2)	36.4 (9.3)	0.00
Age range, n (%), y			0.18[b]			0.00
18–29	611 (25.6)	841 300 (31.6)		528 (24.7)	528 (24.7)	
30–39	728 (30.5)	883 265 (33.2)		654 (30.6)	654 (30.6)	
40–49	1049 (43.9)	939 106 (35.3)		952 (44.6)	952 (44.6)	
Male sex, n (%)	917 (38.4)	1,380,603 (51.8)	0.27[b]	800 (37.5)	737 (34.5)	0.06
US region, n (%)			0.29[b]			0.10
Northeast	213 (8.9)	247,703 (9.3)		200 (9.4)	200 (9.4)	
Midwest	740 (31.0)	682,453 (25.6)		663 (31.1)	623 (29.2)	
South	937 (39.2)	1,051,114 (39.5)		825 (38.7)	910 (42.6)	
West	493 (20.6)	571,517 (21.5)		441 (20.7)	400 (18.7)	
Type of first incident CDI case, n (%)			n/a			n/a
Hospitalization or other inpatient facility	792 (33.2)	n/a		602 (28.2)	n/a	
Outpatient	662 (27.7)	n/a		627 (29.4)	n/a	
Toxin test and antibiotic	934 (39.1)	n/a		905 (42.4)	n/a	
Acquisition status of first incident case,[c] n (%)			n/a			n/a
Healthcare associated	479 (20.1)	n/a		315 (14.8)	n/a	
Community associated	1774 (74.3)	n/a		1710 (80.1)	n/a	
Indeterminate	135 (5.7)	n/a		109 (5.1)	n/a	
Charlson Comorbidity Index, mean (SD)	1.4 (2.3)	0.2 (0.6)	0.74[b]	1.2 (2.0)	0.9 (1.6)	0.17[b]
Charlson Comorbidity Index, n (%)			0.91[b]			0.16[b]
0	1259 (52.7)	2,363,815 (88.7)		1208 (56.6)	1322 (61.9)	
1	459 (19.2)	224,249 (8.4)		421 (19.7)	418 (19.6)	
2	213 (8.9)	50,343 (1.9)		175 (8.2)	174 (8.2)	
3+	457 (19.1)	25,264 (0.9)		330 (15.5)	220 (10.3)	

CDI, *Clostridioides difficile* infection; CDI+, CDI positive; CDI–, CDI negative; n/a, not applicable; Std diff, standardized difference.

[a]Sensitivity analysis was performed on outcomes by excluding those patients without a CDI diagnosis. Included patients met criteria for either 1) inpatient CDI diagnosis, 2) an antibiotic prescription filled within 2 weeks of an outpatient CDI diagnosis, or 3) an antibiotic prescription filled within 2 weeks of a toxin test, plus a subsequent CDI diagnosis within 30 days.

[b]Absolute values >0.1 considered significant

Table S6. Healthcare and Patient Out-of-Pocket Costs at 2 Months by CDI Acquisition and Hospitalization Status and Age Group (Sensitivity Analysis Requiring a CDI Diagnosis[a])

	Healthcare Associated, $						Community Associated, $								
	Hospitalized[b]			Not Hospitalized[b]			Hospitalized[b]			Not Hospitalized[b]			Overall, $		
**Age Group**	**CDI+**	**CDI–**	**Difference**	**CDI+**	**CDI–**	**Difference**	**CDI+**	**CDI–**	**Difference**	**CDI+**	**CDI–**	**Difference**	**CDI+**	**CDI–**	**Difference**
50–64 years	(n=463)	(n=463)		(n=216)	(n=216)		(n=616)	(n=616)		(n=1073)	(n=1073)		(n=2368)	(n=2368)	
Outpatient[c]	13,815 (30,232)	7972 (16,980)	5842	11,438 (25,649)	5825 (17,305)	5613	7646 (19,553)	5309 (14,275)	2337	5320 (18,899)	2736 (8612)	2584	8144 (22,588)	4711 (13,193)	3433
Emergency department	537 (2384)	173 (749)	364	437 (1045)	102 (527)	335	558 (2708)	318 (4393)	241	631 (2122)	101 (609)	530	576 (2271)	172 (2308)	405
Inpatient hospitalization	67,740 (247,794)	8193 (30,719)	59,548	6643 (27,605)	7812 (32,820)	-1169	35,781 (44,740)	2364 (10,348)	33,418	919 (6620)	1418 (12,115)	-499	23,575 (115,208)	3572 (19,612)	20,003
Other inpatient facility[d]	3240 (14,780)	675 (4748)	2566	1910 (6852)	137 (1417)	1773	1332 (6734)	100 (1138)	1231	145 (3045)	15 (506)	129	1220 (8016)	178 (2258)	1042
Prescription	2218 (5217)	1793 (4260)	425	1596 (2938)	1240 (2517)	356	1625 (3330)	1242 (3542)	383	1614 (3846)	999 (3602)	616	1734 (3967)	1239 (3652)	494
Total healthcare	87,551 (265,353)	18,806 (38,947)	68,745	22,024 (41,455)	15,116 (38,663)	6908	46,942 (51,248)	9333 (21,348)	37,609	8629 (21,309)	5269 (16,036)	3360	35,249 (125,281)	9871 (26,354)	25,377
Total out-of-pocket costs[e]	1366 (1979)	644 (1429)	722	663 (1874)	416 (815)	247	2169 (2225)	487 (880)	1682	808 (1088)	415 (855)	392	1258 (1802)	479 (1000)	779
18–49 years	(n=268)	(n=268)		(n=156)	(n=156)		(n=432)	(n=432)		(n=1278)	(n=1278)		(n=2134)	(n=2134)	
Outpatient[c]	12,250 (22,870)	8093 (24,203)	4157	6988 (11,174)	5453 (14,231)	1535	7131 (19,208)	3286 (9387)	3845	3731 (7194)	1513 (5325)	2218	5727 (13,723)	2986 (11,308)	2741
Emergency department	539 (1392)	257 (868)	283	659 (1499)	353 (982)	306	517 (1175)	339 (3315)	178	726 (1923)	139 (645)	587	655 (1705)	210 (1625)	445
Inpatient hospitalization	59,632 (76,633)	5920 (31,368)	53,712	5189 (22,370)	3927 (15,321)	1263	30,885 (37,489)	2859 (17,157)	28,026	728 (5764)	636 (5715)	92	14,556 (38,803)	1990 (14,924)	12,566
Other inpatient facility[d]	922 (5907)	184 (2876)	738	513 (2618)	15 (185)	498	93 (1120)	25 (518)	68	0 (0)	0 (0)	0	172 (2284)	29 (1047)	143
Prescription	1773 (3694)	1633 (5468)	140	1421 (2570)	1346 (3536)	74	1640 (3498)	899 (3350)	742	1334 (3349)	466 (1958)	868	1457 (3377)	764 (3065)	693
Total healthcare	75,116 (80,739)	16,087 (43,716)	59,029	14,769 (26,188)	11,094 (22,847)	3675	40,267 (44,072)	7408 (22,107)	32,860	6518 (11,286)	2753 (8914)	3765	22,568 (43,632)	5979 (21,092)	16,589
Total out-of-pocket costs[e]	1912 (4115)	675 (1622)	1237	457 (748)	393 (812)	65	2311 (2276)	435 (857)	1876	776 (1017)	292 (760)	484	1206 (2071)	376 (942)	830

CDI, *Clostridioides difficile* infection; CDI+, CDI positive; CDI–, CDI negative.

Costs are presented in US dollars after propensity score matching. Costs were adjusted to 2019 US dollars using the medical care component of the Consumer Price Index.

Costs within CDI groups are displayed as means (SD).

[a]Sensitivity analysis was performed on outcomes by excluding those patients without a CDI diagnosis. Included patients met criteria for either 1) inpatient CDI diagnosis, 2) an antibiotic prescription filled within 2 weeks of an outpatient CDI diagnosis, or 3) an antibiotic prescription filled within 2 weeks of a toxin test, plus a subsequent CDI diagnosis within 30 days.

[b]Descriptions of “hospitalized” and “not hospitalized” refer to the CDI+ patients only; matched controls were not necessarily hospitalized. Hospitalized patients were defined as those who were hospitalized at time of CDI diagnosis or hospitalized with a CDI diagnosis code within 60 days post-index.

[c]Excludes emergency department visits.

[d]Skilled nursing facility, inpatient hospice facility, inpatient mental health/chemical dependence facility, or inpatient rehabilitation facility.

[e]Out-of-pocket costs encompassed copays, deductibles, and coinsurance payments.

Table S7. Healthcare Resource Utilization at 2 Months Post-Index by CDI Acquisition and Hospitalization Status in the 50-64-Years Age Group (Sensitivity Analysis Requiring a CDI Diagnosis[a])

	Healthcare Associated						Community Associated								
	Hospitalized[b]			Not Hospitalized[b]			Hospitalized[b]			Not Hospitalized[b]			Overall		
	CDI+ (n=463)	CDI– (n=463)	Difference	CDI+ (n=216)	CDI– (n=216)	Difference	CDI+ (n=616)	CDI– (n=616)	Difference	CDI+ (n=1073)	CDI– (n=1073)	Difference	CDI+ (n=2368)	CDI– (n=2368)	Difference
Any outpatient visits, n (%)[c]	419 (90.5)	422 (91.1)	–0.6	213 (98.6)	183 (84.7)	13.9	572 (92.9)	511 (83.0)	9.9	1073 (100.0)	818 (76.2)	23.8	2277 (96.2)	1934 (81.7)	14.5
Mean number of outpatient visits per patient (SD)[c]	17.8 (23.7)	9.8 (11.9)	8.0	14.8 (13.9)	7.2 (9.7)	7.6	10.4 (14.2)	6.8 (10.7)	3.7	9.1 (12.1)	4.5 (6.0)	4.6	11.7 (16.1)	6.4 (9.3)	5.3
Any ED visits, n (%)	80 (17.3)	42 (9.1)	8.2	52 (24.1)	12 (5.6)	18.5	117 (19.0)	43 (7.0)	12.0	314 (29.3)	58 (5.4)	23.9	563 (23.8)	155 (6.5)	17.2
Mean number of ED visits per patient (SD)	0.3 (0.9)	0.1 (0.4)	0.2	0.3 (0.7)	0.1 (0.2)	0.3	0.3 (0.7)	0.1 (0.5)	0.2	0.4 (0.6)	0.1 (0.3)	0.3	0.3 (0.7)	0.1 (0.4)	0.2
Inpatient hospitalization, n (%)	463 (100.0)	78 (16.8)	83.2	28 (13.0)	29 (13.4)	–0.5	616 (100.0)	48 (7.8)	92.2	41 (3.8)	39 (3.6)	0.2	1148 (48.5)	194 (8.2)	40.3
Mean number of inpatient days per patient (SD)	15.4 (14.7)	1.8 (6.4)	13.6	1.3 (4.7)	1.3 (4.4)	0.0	9.7 (10.8)	0.6 (2.6)	9.1	0.3 (2.9)	0.4 (3.1)	0.0	5.8 (10.8)	0.8 (4.0)	5.0
Any outpatient prescription, n (%)	391 (84.4)	425 (91.8)	–7.3	206 (95.4)	193 (89.4)	6.0	569 (92.4)	545 (88.5)	3.9	1060 (98.8)	889 (82.9)	15.9	2226 (94.0)	2052 (86.7)	7.3
Mean number of outpatient prescriptions per patient (SD)	9.9 (8.5)	9.3 (7.6)	0.6	9.3 (8.0)	8.7 (7.8)	0.6	9.0 (7.2)	7.5 (6.8)	1.5	7.6 (5.9)	5.8 (6.1)	1.8	8.6 (7.1)	7.2 (6.9)	1.4

CDI, *Clostridioides difficile* infection; CDI+, CDI positive; CDI–, CDI negative; ED, emergency department.

Values presented after propensity score matching. Numbers and days spent in other inpatient facilities (skilled nursing facility, inpatient hospice facility, inpatient mental health/chemical dependence facility, or inpatient rehabilitation facility) are not shown in order to maintain patient de-identification due to small cell counts.

[a]Sensitivity analysis was performed on outcomes by excluding those patients without a CDI diagnosis. Included patients met criteria for either 1) inpatient CDI diagnosis, 2) an antibiotic prescription filled within 2 weeks of an outpatient CDI diagnosis, or 3) an antibiotic prescription filled within 2 weeks of a toxin test, plus a subsequent CDI diagnosis within 30 days.

[b]Descriptions of “hospitalized” and “not hospitalized” refer to the CDI+ patients only; matched controls were not necessarily hospitalized. Hospitalized patients were defined as those who were hospitalized at time of CDI diagnosis or hospitalized with a CDI diagnosis code within 60 days post-index.

[c]Excludes ED visits.

Table S8. Healthcare Resource Utilization at 2 Months Post-Index by CDI Acquisition and Hospitalization Status in the 18–49 Years Age Group (Sensitivity Analysis Requiring a CDI Diagnosis[a])

	Healthcare Associated						Community Associated								
	Hospitalized[b]			Not Hospitalized[b]			Hospitalized[b]			Not Hospitalized[b]			Overall		
	CDI+ (n=268)	CDI– (n=268)	Difference	CDI+ (n=156)	CDI– (n=156)	Difference	CDI+ (n=432)	CDI– (n=432)	Difference	CDI+ (n=1278)	CDI– (n=1278)	Difference	CDI+ (n=2134)	CDI– (n=2134)	Difference
Any outpatient visits, n (%)[c]	236 (88.1)	223 (83.2)	4.9	156 (100.0)	131 (84.0)	16.0	405 (93.8)	326 (75.5)	18.3	1278 (100.0)	810 (63.4)	36.6	2075 (97.2)	1490 (69.8)	27.4
Mean number of outpatient visits per patient (SD)[c]	12.7 (12.7)	7.8 (10.8)	4.9	11.6 (12.4)	5.9 (6.9)	5.7	9.4 (10.2)	4.9 (7.4)	4.5	7.7 (6.3)	3.2 (5.5)	4.5	8.9 (8.9)	4.3 (7.1)	4.6
Any ED visits, n (%)	62 (23.1)	33 (12.3)	10.8	50 (32.1)	28 (17.9)	14.1	112 (25.9)	39 (9.0)	16.9	465 (36.4)	95 (7.4)	29.0	689 (32.3)	195 (9.1)	23.1
Mean number of ED visits per patient (SD)	0.4 (0.9)	0.2 (0.6)	0.2	0.5 (0.9)	0.3 (0.7)	0.2	0.3 (0.7)	0.1 (0.7)	0.2	0.5 (0.7)	0.1 (0.4)	0.4	0.4 (0.7)	0.1 (0.5)	0.3
Inpatient hospitalization, n (%)	268 (100.0)	31 (11.6)	88.4	21 (13.5)	15 (9.6)	3.8	431 (99.8)	35 (8.1)	91.7	41 (3.2)	32 (2.5)	0.7	761 (35.7)	113 (5.3)	30.4
Mean number of hospitalizations per patient (SD)	1.6 (0.9)	0.2 (0.5)	1.4	0.2 (0.4)	0.1 (0.4)	0.0	1.2 (0.5)	0.1 (0.3)	1.1	0.0 (0.2)	0.0 (0.2)	0.0	0.5 (0.8)	0.1 (0.3)	0.4
Mean number of inpatient days per patient (SD)	16.5 (16.4)	1.6 (7.1)	14.9	1.4 (5.2)	1.2 (5.2)	0.2	8.1 (8.6)	0.7 (3.3)	7.4	0.2 (1.1)	0.2 (1.2)	0.0	3.9 (9.1)	0.5 (3.4)	3.4
Any outpatient prescription, n (%)	235 (87.7)	219 (81.7)	6.0	152 (97.4)	133 (85.3)	12.2	405 (93.8)	341 (78.9)	14.8	1256 (98.3)	878 (68.7)	29.6	2048 (96.0)	1571 (73.6)	22.4
Mean number of outpatient prescriptions per patient (SD)	9.5 (8.6)	6.6 (7.4)	2.9	7.3 (6.3)	6.4 (6.8)	0.9	8.1 (6.3)	4.8 (5.5)	3.3	5.8 (5.5)	3.5 (4.5)	2.3	6.9 (6.3)	4.4 (5.5)	2.5

CDI, *Clostridioides difficile* infection; CDI+, CDI positive; CDI–, CDI negative; ED, emergency department.

Values presented after propensity score matching. Numbers and days spent in other inpatient facilities (skilled nursing facility, inpatient hospice facility, inpatient mental health/chemical dependence facility, or inpatient rehabilitation facility) are not shown in order to maintain patient de-identification due to small cell counts.

[a]Sensitivity analysis was performed on outcomes by excluding those patients without a CDI diagnosis. Included patients met criteria for either 1) inpatient CDI diagnosis, 2) an antibiotic prescription filled within 2 weeks of an outpatient CDI diagnosis, or 3) an antibiotic prescription filled within 2 weeks of a toxin test, plus a subsequent CDI diagnosis within 30 days.

[b]Descriptions of "hospitalized" and "not hospitalized" refer to the CDI+ patients only; matched controls were not necessarily hospitalized. Hospitalized patients were defined as those who were hospitalized at time of CDI diagnosis or hospitalized with a CDI diagnosis code within 60 days post-index.

[c]Excludes ED visits.
